# Cattle selectivity by leopards suggests ways to mitigate human–leopard conflict

**DOI:** 10.1002/ece3.4351

**Published:** 2018-07-16

**Authors:** Igor Khorozyan, Siavash Ghoddousi, Mobin Soufi, Mahmood Soofi, Matthias Waltert

**Affiliations:** ^1^ Workgroup on Endangered Species J.F. Blumenbach Institute of Zoology and Anthropology Georg‐August‐Universität Göttingen Göttingen Germany; ^2^ APM Co. Tehran Iran; ^3^ Department of the Environment Faculty of Fishery and Environment Gorgan University of Agricultural Sciences and Natural Resources Gorgan Iran

**Keywords:** carnivore, human–wildlife conflict, Hyrcanian forest, Iran, livestock depredation, *Panthera pardus*, predator

## Abstract

Addressing widespread livestock losses to carnivores requires information on which livestock categories are preferentially selected. We analyzed an individual‐based database of cattle grazing in forest (*n* = 932) and having been killed (*n* = 70) by leopards (*Panthera pardus*) in the Hyrcanian forest, Iran. We calculated Jacobs’ selectivity index for cattle age, sex, and coloration across four scales: the study area as a whole, three sites, nine villages, and 60 cattle owners. Naturally colored cattle were significantly preferred by leopards at all scales in comparison with black and black‐and‐white cattle, and there was also a preference for males and juveniles at the study area level. More research is needed to see whether cattle losses would decrease if the share of naturally colored individuals in local holdings was reduced and males and juveniles had limited access to forest. We conclude that phenotypic and biologic characteristics of livestock can affect depredation and appeal for more research in this direction, particularly within the predator–prey framework.

## 1 INTRODUCTION

Conflicts between local people and mammalian carnivores are widespread because of depredation on domestic animals, especially livestock, and occasional attacks on humans (Eklund, Lopez‐Bao, Tourani, Chapron, & Frank, 2017; Loveridge, Wang, Frank, & Seidensticker, 2010). Such conflicts pose a challenge for rural development and biodiversity conservation as many carnivores are officially protected and act as conservation flagships, but financial and social losses from depredation fuel intolerance to wildlife and conservation in general. Conflicts are particularly common in developing countries where local communities are poor and have limited opportunities for alternative livelihoods to compensate losses (Loveridge et al., 2010; Suryawanshi, Bhatnagar, Redpath, & Mishra, 2013).

Selective killing of livestock by carnivores has high practical importance as it may identify most vulnerable livestock species or intraspecific categories and potentially help reduce conflicts by targeting these high‐risk species or categories. Carnivores may give preference to particular livestock species and take proportionally higher numbers than available (Chetri, Odden, & Wegge, 2017; Elbroch & Wittmer, 2013) or, alternatively, avoid or prey them opportunistically (Ghoddousi et al., 2016; Lyngdoh et al., 2014; Suryawanshi et al., 2013).

Livestock selectivity is not only about high‐risk species as intraspecific livestock categories based on individual traits (age, sex, coloration, and breed) also can determine depredation patterns. Juvenile individuals of livestock are more prone to depredation than adults because of their weakly developed antipredator behavior (Azevedo & Murray, 2007; Michalski, Boulhosa, Faria, & Peres, 2006; Odden, Herfindal, Linnell, & Andersen, 2008). Livestock breeds are also important as heavier and less agile breeds or those with poor defensive capacities can be preferentially taken by carnivores (Eklund et al., 2017; Landa, Gudvangen, Swenson, & Røskaft, 1999; Quigley et al., 2015). Sex‐based selectivity by carnivores has been studied in wild prey, but not in livestock (Anderson & Lindzey, 2003; Karanth & Sunquist, 1995; Majumder, Sankar, Qureshi, & Basu, 2013).

Livestock selectivity studies require a consideration of different scales so as to determine how selectivity patterns may differ between study areas as a whole, individual study sites, villages, and households. Patterns that appear at all or most scales deserve special attention as they demonstrate strong effects on livestock irrespective of individual heterogeneity and scale‐dependent factors such as livestock numbers. Depredation is an intrinsically spatial phenomenon because livestock losses can be low at large scales, but detrimental for villages and households (Khorozyan, Soofi, Hamidi, Ghoddousi, & Waltert, 2015; Michalski et al., 2006); losses are unevenly distributed in particular “hotspot” villages and households (Heinonen & Travis, 2015); and management decisions must be essentially beneficial for livestock owners, not only in general (Heinonen & Travis, 2015).

**Figure 1:**
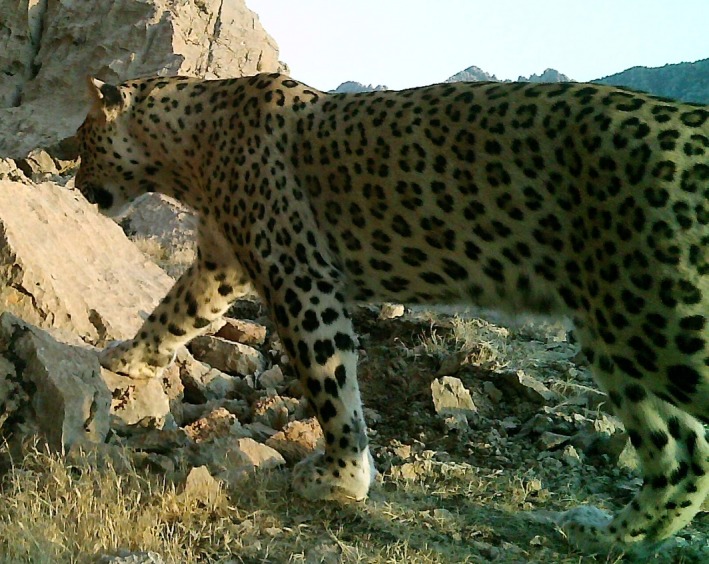
The Persian leopard (*Panthera pardus saxicolor*). Credits: Nature Iraq/Goldman Environmental Foundation/General Consulate of Germany in Erbil, Kurdistan

In this study, we determine selectivity of cattle (*Bos taurus*) by leopards (*Panthera pardus*) (Figure 1) in the Hyrcanian forest of northern Iran at different scales, from individual households to villages, study sites, and the whole study area. The scales we used in this study (from households to study area) are relevant to livestock loss estimation and mitigation, but they can be dissimilar to spatial scales commonly used in ecological predator–prey studies (Miller, Jhala, Jena, & Schmitz, 2015; Trainor & Schmitz, 2014). We test three hypotheses: (a) Male cattle are preferred by leopards because males have a larger body mass, and leopards in Iran are among the biggest; (b) juvenile cattle (calves and heifers) are preferred by leopards because they are easy to catch and thus are highly vulnerable to depredation; and (c) differently colored cattle are neither preferred nor avoided by leopards and taken according to the cattle availability. Finally, we suggest practical solutions to mitigation of human–leopard conflict in the Hyrcanian forest. This study is novel in terms of research findings and practical applications, in Iran and beyond. Also, this study is timely and important because Iran is the stronghold country for the globally endangered leopard subspecies in the Middle East, the Persian leopard (*Panthera pardus saxicolor*) (Figure 1; Ebrahimi, Farashi, & Rashki, 2017; Khorozyan, 2014), the Hyrcanian forest represents the most continuous and most suitable habitat for leopard (Ebrahimi et al., 2017; Farashi & Shariati, 2017; Farashi, Shariati, & Hosseini, 2017), and retaliatory anthropogenic mortality from depredation is among the main threats to leopard survival in Iran and the region (Ghoddousi et al., 2017; Soofi et al., 2018).

## 2 MATERIALS AND METHODS

**Figure 2:**
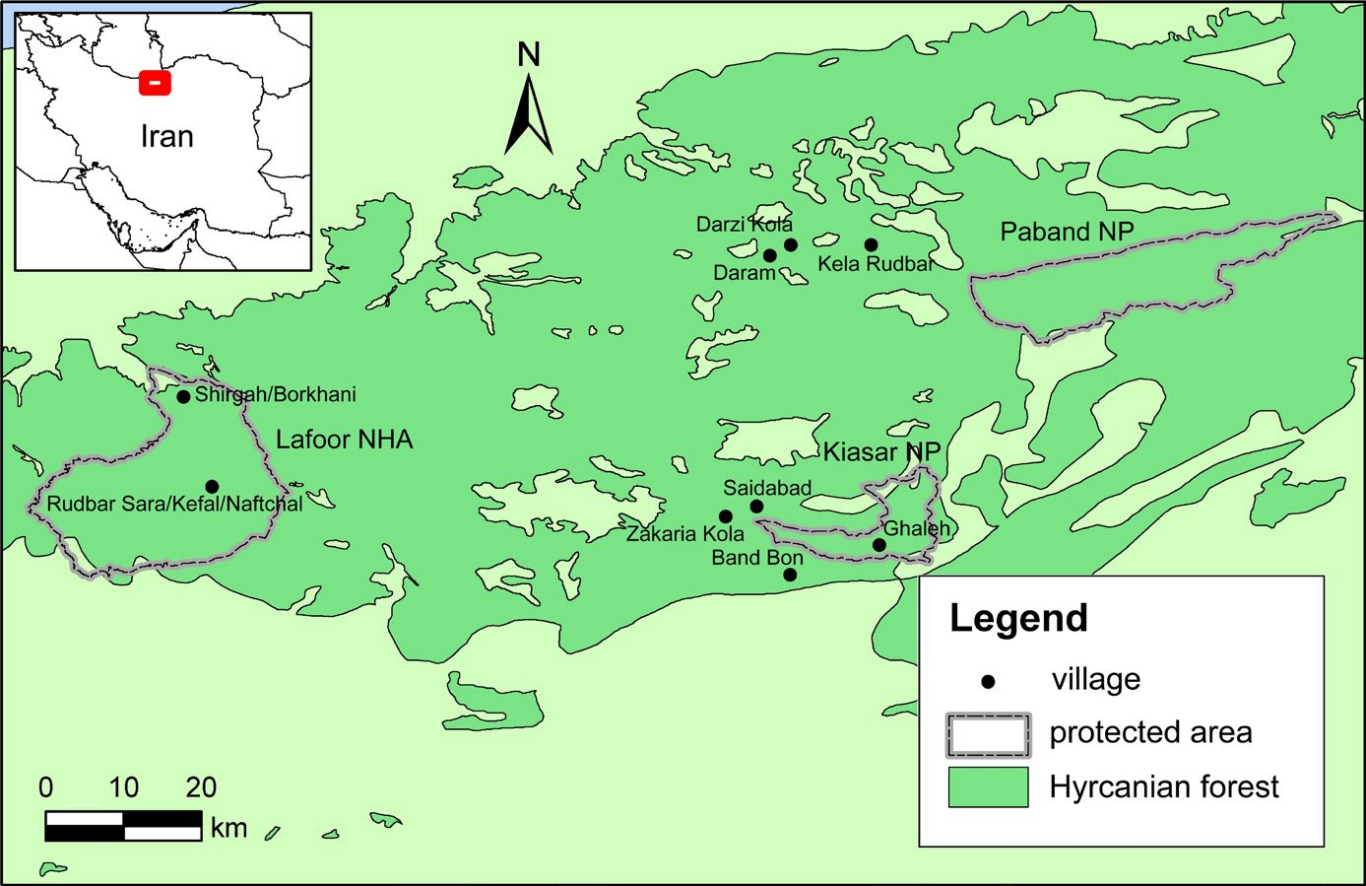
Location of surveyed villages near Paband and Kiasar National Parks (NP) and Lafoor No‐Hunting Area (NHA) in Mazandaran Province, northern Iran

We surveyed nine villages near and inside Paband and Kiasar National Parks (NP) and Lafoor No‐Hunting Area (NHA) in Mazandaran Province, northern Iran (Figure 2). The main landscape is the Hyrcanian forest, also known as the Caspian forest, a Tertiary relict primary deciduous temperate forest of very rich biodiversity and high levels of species endemism (Akhani, Djamali, Ghorbanalizadeh, & Ramezani, 2010; Sagheb‐Talebi, Sajedi, & Pourhashemi, 2014). It spans throughout the provinces of Golestan, Mazandaran, and Gilan on the northern slopes of the Alborz Ridge fringing the southern coast of the Caspian Sea and is part of the Caucasus–Anatolian–Hyrcanian temperate forest ecoregion and the Caucasus biodiversity hotspot (Marchese, 2015; Olson & Dinerstein, 2002; http://www.globalspecies.org). The existing network of protected areas is inadequate to achieve conservation goals, and anthropogenic threats such as infrastructure development, grazing, logging, wildlife poaching, and wood collection are widespread (Mehri, Salmanmahiny, Mirkarimi, & Rezaei, 2014; Noack, Manthey, Ruitenbeek, & Mohadjer, 2010; Sadeghian, 2016; Soofi et al., 2018). As a result, the coverage of the Hyrcanian forest has halved during the past decades from 3.6 to 1.8 million hectares (Akhani et al., 2010; Sagheb‐Talebi et al., 2014).

The Hyrcanian forest has been used for grazing of about 4 million heads of livestock, and grazing is among the main threats to large mammals, particularly leopard (Sagheb‐Talebi et al., 2014; Soofi et al., 2018). Cattle are the dominant livestock in forested areas bred for milk, dairy products, beef, and breeding (Babrgir, Farhadinia, & Moqanaki, 2017; Noack et al., 2010). Mazandarani is the main local cattle breed of low introgression and better genetic quality than other native breeds (Karimi, Koshkoiyeh, Fozi, Porto‐Neto, & Gondro, 2016; Karimi, Strucken et al., 2016). Stud cattle and cows with juveniles are kept in village corrals, and a majority of cattle graze in forest, usually unattended by shepherds and dogs (this study; Babrgir et al., 2017; Ghoddousi et al., 2016; Khorozyan et al., 2015; Khorozyan et al., 2017). Sometimes, cows with juveniles are also left grazing in forest. Free grazing is practiced from March‐April to October‐November and also during warm winters. Cattle may return to corrals every evening (Babrgir et al., 2017; Noack et al., 2010), but often they stay overnight in forest (this study). Many owners leave cattle in forest all the time during the warm season and drive them to corrals in autumn and winter (this study). As a result of lax practices, forest‐grazing cattle suffer from high depredation by leopards throughout the Hyrcanian forest, usually during the warm season (Babrgir et al., 2017; Ghoddousi et al., 2016; Khorozyan et al., 2015, 2017).

We conducted structured interview surveys in Persian among three to four cattle owners who lost cattle to leopards and three to four cattle owners without such losses in each of nine villages (Figure 2). We selected the households randomly and interviewed the oldest person from each household. We assumed that these households are independent because each owner grazes his cattle in a particular place, which is rarely marked or fenced, but is well known to all villagers. We selected these villages because they experienced the highest levels of leopard depredation on cattle in Mazandaran Province, as the provincial office of Department of Environment (DoE) recorded. We used a standard questionnaire protocol form and surveyed villages under the guidance of park rangers and other authorized representatives of DoE. All respondents were informed about their anonymity and study aims and gave their consent to participate. No animal handling was conducted.

We recorded gender and age of the respondents and asked them about the numbers, sex, age, and coloration of cattle grazing in forest and lost to leopards during the previous year from September 2016 to September 2017. We established the individual‐based database with sex, age, and color of each individual cattle. Sex and coloration were unrelated; that is, owners did not strive to breed males and females of certain colors (our observation). We also recorded seasons of cattle kills by leopards. We asked owners how often do they or their shepherds attend cattle in forest (never, rarely, and daily) and how often do cattle stay overnight in corrals (never, sometimes, and always). We categorized cattle age as adult (>2 years) and juvenile (<2 years) and recorded cattle coloration as black, black‐and‐white, red, red‐and‐white, yellow‐and‐white, and gray. We considered black and black‐and‐white coloration as “exotic” because it does not occur in leopard main prey in the Hyrcanian forest, which are the wild boar (*Sus scrofa*), bezoar goat (*Capra aegagrus*), Caspian red deer (*Cervus elaphus maral*), and roe deer (*Capreolus capreolus*) (Ghoddousi et al., 2016, 2017; Soofi et al., 2018). In contrast, we considered red, red‐and‐white, yellow‐and‐white, and gray coloration as “natural” because it resembled closely the coloration of leopard main prey in the Hyrcanian forest and Iran in general.

The gray wolf (*Canis lupus*) is the only other large carnivore in the Hyrcanian forest which may take cattle and its kills can be misidentified as those of leopard. Owners correctly attributed 70 cattle kills to leopards by indicating tracks with predation signs, scrapes, or sounds (40, 57.1%), throat bites (*n* = 13, 18.6%), leopard observations (*n* = 12, 17.1%), absence of other large carnivores (*n* = 3, 4.3%), and confirmation of leopard kills by DoE experts (*n* = 2, 2.9%).

We calculated Jacobs’ selectivity index (*I*_*j*_) to measure the selectivity of cattle by leopards (Ghoddousi et al., 2016; Hayward et al., 2006; Lyngdoh et al., 2014) as follows:

$$I_{j}=\frac{r_{i}-p_{i}}{r_{i}+p_{i}-2r_{i}p_{i}}$$

where *r*_*i*_ is the proportion of the *i*th cattle category in killed cattle and *p*_*i*_ is this proportion for cattle grazing in forest. Values of *I*_*j*_ vary from +1 to −1, indicating +1 as strong preference, −1 as strong avoidance, and 0 meaning that cattle are killed proportionally to their availability (no selectivity). We measured *I*_*j*_ for sex, age, separate natural colors, and all naturally colored cattle.

We considered *I*_*j*_ at four scales: the study area as a whole, three individual study sites (Paband NP, Kiasar NP, and Lafoor NHA), nine villages (Figure 2), and 60 cattle owners (respondents). For this, we aggregated data from cattle owners over their villages, data from villages over study sites, and data from study sites over the whole study area. Except for the study area, we calculated the standard error (*SE*) for *I*_*j*_ as a measure of its variation. We studied the difference of *I*_*j*_ from zero by Shapiro–Wilk normality test and one‐sample *t*‐test (Hayward et al., 2006; Lyngdoh et al., 2014). We checked the relationships between cattle losses, cattle attendance by owners or shepherds, and cattle stay in night corrals by Kruskal–Wallis test, used Spearman's correlation *ρ* to find the association between numbers of cattle grazing in forest and cattle losses, and applied chi‐square test to find how cattle losses differ between seasons. We used IBM SPSS Statistics v. 23 at significance level *p* = 0.05 for statistical analysis.

## 3 RESULTS

**Figure 3:**
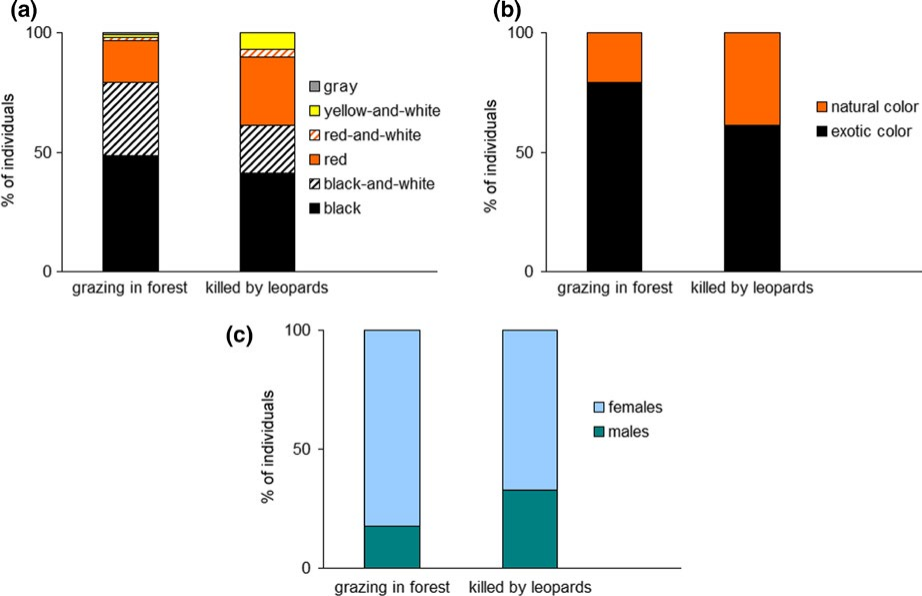
The proportions of separate natural colors (a), exotic and natural coloration (b), and sex (c) among cattle grazing in forest and killed by leopards in the study area

We surveyed 60 cattle owners from nine villages: three villages near Paband NP (*n* = 19 owners), four near Kiasar NP (*n* = 29), and two near Lafoor NHA (*n* = 12) (Figure 2; Supporting Information Appendix S1: Tables S1 and S2). Most (*n* = 58, 96.7%) of them were men of the mean age 52.8 ± 2.1 years. Sixty owners had 932 heads of cattle grazing in forest (15.5 ± 1.9 heads/owner), and 33 owners of them incurred losses of 70 cattle to leopards in September 2016 to September 2017, on average 2.1 ± 0.2 heads per affected owner and year. The distribution of sex, age, and color categories among forest‐grazing and killed cattle is given graphically in Figure 3 and in numbers in Supporting Information Appendix S1: Tables S1 and S2 .

**Figure 4:**
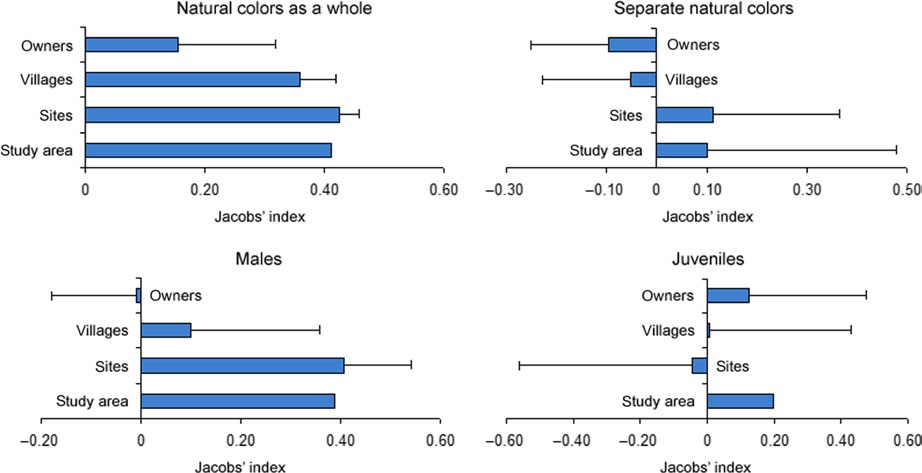
Cattle selectivity for natural coloration as a whole, separate natural colors, males and juveniles by leopards in the study area, sites, villages, and owners. Error bars indicate the standard error

Leopard significantly preferred cattle of natural coloration in study area (*I*_*j*_ = 0.41), sites (*I*_*j*_ = 0.42 ± 0.03, *t* = 12.612, *p* = 0.006), and villages (*I*_*j*_ = 0.36 ± 0.06, *t* = 5.954, *p* < 0.001) (Figure 4). Preference of naturally colored cattle was not evident in individual owners (*I*_*j*_ = 0.15 ± 0.16, *t* = 0.940, *p* = 0.356) because some owners had very few of such cattle and none of them were lost to leopards, yielding *I*_*j*_ = −1 (Figure 4). Yet, a majority of owners experienced a significant positive selection of cattle with natural coloration (*I*_*j*_ = 0.73 ± 0.06, *t* = 11.601, *p* < 0.001). When analyzing different natural colors separately, leopards did not select cattle of single natural colors (*I*_*j*_ varied from −0.10 ± 0.16 to 0.11 ± 0.25, *t* from −0.614 to 0.436, *p* > 0.05). Male cattle, that is, bulls and calves, were positively selected in the study area (*I*_*j*_ = 0.39), but not significantly in sites (*I*_*j*_ = 0.41 ± 0.14, *t* = 2.965, *p* = 0.097), villages (*I*_*j*_ = 0.10 ± 0.26, *t* = 0.384, *p* = 0.711), and owners (*I*_*j*_ = −0.01 ± 0.17, *t* = −0.055, *p* = 0.957). There was some preference for juveniles, that is, calves and heifers, in the study area (*I*_*j*_ = 0.20), but not in sites (*I*_*j*_ = −0.05 ± 0.52, *t* = −0.090, *p* = 0.936), villages (*I*_*j*_ = 0.01 ± 0.42, *t* = 0.014, *p* = 0.989), and owners (*I*_*j*_ = 0.13 ± 0.35, *t* = 0.357, *p* = 0.732) (Figure 4).

Attendance of grazing cattle by owners or shepherds (Kruskal–Wallis *χ* ^2^ = 3.056, *p* = 0.217) and cattle staying in night corrals (*χ* ^2^ = 5.585, *p* = 0.061) did not affect cattle losses to leopards. Also, losses did not correlate with numbers of cattle grazing in forest (Spearman's *ρ* = 0.227, *p* = 0.081). Most cattle were killed during the warm season from spring to autumn (*n* = 64) in comparison with winter (*n* = 5), and this difference was significant (*χ* ^2^ = 50.449, *p* < 0.001). There was no difference between cattle losses in spring (*n* = 15), summer (*n* = 19), and autumn (*n* = 30) (*χ* ^2^ = 5.665, *p* = 0.059).

## 4 DISCUSSION

This study demonstrates that naturally colored (red, red‐and‐white, yellow‐and‐white, and gray) cattle were significantly preferred vis‐à‐vis black and black‐and‐white cattle by leopards across our study area in the Hyrcanian forest and also at site, village, and household levels (Figures 3 and 4). To our knowledge, this is the first description of selective depredation by carnivores based on livestock coloration. This selection could be caused by the resemblance of naturally colored individuals to wild prey species which might provoke hunting behavior in leopards. However, felids are dichromatic and shortsighted (Clark & Clark, 2016; Jacobs, 1993; Melin, Kline, Hiramatsu, & Caro, 2016) and it is generally acknowledged that predators, including felids, recognize prey from their contrast and brightness against a backdrop rather than from coloration per se (Jacobs, 1993; Ortolani, 1999). This implies that naturally colored livestock may be more prone to predator attacks on a complex background of dense forest vegetation (Melin et al., 2016). Wild species tend to be darker, even to become black, in forests because they survive by concealing from predators in vegetation (Caro, 2005). Although coloration of livestock and other domestic animals is driven by artificial selection and not by natural selection, black cattle also can be killed less frequently due to their concealment. Similar to zebras (*Equus* spp.) that can successfully merge with woodland vegetation and become less detectable for African predators (Melin et al., 2016), black‐and‐white cattle also may benefit from hiding in forest. Black‐and‐white cattle are unlikely to use their coloration for warning (aposematism; Caro, 2009; Stankowich, Caro, & Cox, 2011) because they lack defensive behavior of aposematic species such as local Indian crested porcupine (*Hystrix indica*) and Southwest Asian badger (*Meles canescens*). Therefore, we assume that naturally colored cattle were preferred by leopards because they were more conspicuous in the dense Hyrcanian forest. It is unlikely that naturally colored individuals would have some other characteristics, such as different antipredator behavior or morphological features, making them more prone to predation. The reason is that all cattle in sampled households belonged to the Mazandarani breed, which is diverse in colors but uniform in behavior and morphology (Karimi, Koshkoiyeh et al., 2016; Karimi, Strucken et al., 2016). Male and juvenile cattle were more susceptible to predation, but this was related to their small body size (juveniles), behavior (males), and generally low numbers and not to their colors which were diverse. Although naturally colored cattle and wild prey have similar coloration, they have different body sizes and morphology, but this difference is unlikely to be important as leopards are very catholic in their diet (Hayward et al., 2006) and large size of leopards in Iran widens the range of potential prey, from small to very large (Khorozyan, 2014). Conspicuousness of naturally colored prey and concealment of black and black‐and‐white cattle could vary between the dark and light times of the day, but we could not test this because of a limited availability of cattle at night in places where they are kept overnight in corrals.

We did not find evidence for other factors such as herding, staying in night corrals, number of cattle grazing in forest, and seasons affecting cattle depredation by leopards as strongly as cattle coloration did. We assumed that sampled villages were equally prone to depredation as they represented the “hotspots” of the highest depredation in Mazandaran Province. We also assumed that the effect of depredation on households was random as we did not find differences in husbandry practices and grazing patterns between households with losses and those without losses.

Local leopards also preferred to kill male and juvenile individuals of cattle, but only at the largest scale, the whole study area (Figure 4). Preferences of male cattle can be explained by the fact that males may leave females during grazing (this study; Bouissou, Boissy, Le Neindre, & Veissier, 2001) and a much lower number of males in grazing herds make them more vulnerable to predation when they graze apart. The fact that leopards in Iran are among the largest in the world (Khorozyan, 2014) also contributes to selective killing of male cattle. Juveniles of cattle are often preferred because they are much easier to catch than adults (Azevedo & Murray, 2007; Michalski et al., 2006). Lack of evidence of selective depredation on males and juveniles at fine scales was caused by low numbers of forest‐grazing males and juveniles which led to a high contrast (strong preference vs. strong avoidance) of their selectivity indices between sites, villages, and households.

With this study, we appeal for more research and practical applications on phenotypic and biologic characteristics of livestock as the determinants of depredation. We suggest that these characteristics may play a certain role in livestock depredation, but apart from selective preference of juveniles (Azevedo & Murray, 2007; Michalski et al., 2006; Odden et al., 2008), little is known about selectivity of sex and coloration of livestock by predators. Livestock breeds may also affect depredation rates (Eklund et al., 2017; Landa et al., 1999), but we could not study this aspect as only one cattle breed has been kept in our sampled villages. Potentially, livestock selectivity studies can be considered within the predator–prey framework if kill sites are explicitly known for making inferences over the fine‐scale site‐specific relationships (Miller et al., 2015; Trainor & Schmitz, 2014). Locally, the results of this study have strong practical implications for potential reduction of cattle losses to leopard depredation in the Hyrcanian forest. More research is needed to see whether cattle losses would decrease if we recommend to local cattle owners to reduce the share of naturally colored individuals in local holdings and to limit the access of males and juveniles in forest. Although preference of males and juveniles was not supported at fine scales, loss of a single male or juvenile by a household can undermine the family income relying on breeding and replenishment of less productive cattle. As the Hyrcanian forest is a biodiversity hotspot and a key area for leopard conservation (Ebrahimi et al., 2017; Farashi & Shariati, 2017; Farashi et al., 2017) which needs better protection (Mehri et al., 2014), we hope that this practice will reduce conflicts and improve conservation status of this region.

## AUTHOR CONTRIBUTIONS

IK and MW conceived and designed the study. IK, SG, MS, and MahS collected and analyzed the data. IK wrote the manuscript. All authors read, revised, and approved the final version of the manuscript.

## DATA ACCESSIBILITY STATEMENT

Data available from the Dryad Digital Repository: https://doi.org/10.5061/dryad.1nc461g.
