# Supplementary material for: Cattle selectivity by leopards suggests ways to mitigate human–leopard conflict
Source: Ecol Evol. 2018 Jul 16;8(16):8011–8. doi: 10.1002/ece3.4351 (PMC6144960; doi:10.1002/ece3.4351)
Supplement: Supplementary file 1 [file ECE3-8-8011-s001.doc]

**APPENDIX S1**

**Table S1.** Distribution of sexes (M, males and F, females) and coloration in forest-grazing cattle of the studied villages.

| **Area** | **Village** | **No. owners** | **M (bulls and calves)** | **F (cows and heifers)** | **Black** | **Black-and-white** | **Red** | **Red-and-white** | **Yellow-and-white** | **Grey** |
| --- | --- | --- | --- | --- | --- | --- | --- | --- | --- | --- |
| Paband NP | Kela Rudbar | 7 | 27 | 107 | 87 | 21 | 19 | 0 | 0 | 7 |
|  | Darzi Kola | 6 | 17 | 80 | 69 | 10 | 18 | 0 | 0 | 0 |
|  | Daram | 6 | 13 | 43 | 31 | 3 | 18 | 4 | 0 | 0 |
| Kiasar NP | Saidabad | 7 | 12 | 76 | 42 | 36 | 9 | 1 | 0 | 0 |
|  | Band Bon | 7 | 15 | 34 | 22 | 8 | 19 | 0 | 0 | 0 |
|  | Ghaleh | 8 | 14 | 102 | 37 | 62 | 16 | 1 | 0 | 0 |
|  | Zakaria Kola | 7 | 4 | 52 | 23 | 25 | 7 | 1 | 0 | 0 |
| Lafoor NHA | Rudbar Sara/Kefak/Naftchal | 6 | 28 | 166 | 100 | 37 | 41 | 4 | 12 | 0 |
|  | Shirgah/Borkhani | 6 | 35 | 107 | 42 | 84 | 15 | 1 | 0 | 0 |
| **Total** |  | **60** | **165** | **767** | **453** | **286** | **162** | **12** | **12** | **7** |

**Table S2.** Distribution of sexes (M, males and F, females) and coloration in cattle killed by leopards in the studied villages.

| **Area** | **Village** | **No. owners** | **M (bulls and calves)** | **F (cows and heifers)** | **Black** | **Black-and-white** | **Red** | **Red-and-white** | **Yellow-and-white** | **Grey** |
| --- | --- | --- | --- | --- | --- | --- | --- | --- | --- | --- |
| Paband NP | Kela Rudbar | 4 | 4 | 2 | 4 | 0 | 1 | 1 | 0 | 0 |
|  | Darzi Kola | 3 | 1 | 4 | 3 | 0 | 2 | 0 | 0 | 0 |
|  | Daram | 3 | 4 | 1 | 1 | 1 | 3 | 0 | 0 | 0 |
| Kiasar NP | Saidabad | 3 | 5 | 0 | 2 | 2 | 1 | 0 | 0 | 0 |
|  | Band Bon | 3 | 1 | 8 | 4 | 1 | 4 | 0 | 0 | 0 |
|  | Ghaleh | 4 | 0 | 6 | 2 | 2 | 1 | 1 | 0 | 0 |
|  | Zakaria Kola | 4 | 0 | 7 | 2 | 2 | 3 | 0 | 0 | 0 |
| Lafoor NHA | Rudbar Sara/Kefak/Naftchal | 5 | 4 | 15 | 8 | 2 | 4 | 0 | 5 | 0 |
|  | Shirgah/Borkhani | 4 | 4 | 4 | 3 | 4 | 1 | 0 | 0 | 0 |
| **Total** |  | **33** | **23** | **47** | **29** | **14** | **20** | **2** | **5** | **0** |
